# Pets, intimacy, and mental health: A study with older couples

**DOI:** 10.1192/j.eurpsy.2025.1765

**Published:** 2025-08-26

**Authors:** S. von Humboldt, S. Silva, I. Leal

**Affiliations:** 1 William James Center for Research; 2 ISPA – Instituto Universitário, Lisbon, Portugal

## Abstract

**Introduction:**

Pet companionship plays a pivotal role in the lives of older couples, significantly influencing their intimate relationships, by fostering deeper connections and enhancing mental well-being.

**Objectives:**

This study has two aims: (1) to explore the impact of pet companionship on the intimacy of older couples; and (2) to examine its influence on their mental health.

**Methods:**

This study included 223 older couples aged between 65 and 94 years. To explore the multifaceted role of pets in their relationship, content analysis was conducted on all interviews.

**Results:**

Their experiences shed light on the significance of this bond for promoting healthy and fulfilling intimate relationships, with the following themes: (1) contributing to emotional well-being (87.3%), (2) improving mental health in later life (83.1%), (3) fostering a deep relationship between couples (78.7%), (4) facilitating the formation of new emotional connections with others (72.1%), and (5) boosting physical and sensory functions (67.2%).

**Conclusions:**

The findings emphasize the diverse advantages of pet companionship among older adults. These include enhancing emotional well-being, bolstering mental health, fostering interpersonal bonds, and improving physical capacities. These insights underscore the potential for integrating pet interventions into comprehensive strategies aimed at promoting the overall quality of life for older individuals.

Keywords: Pet companionship; intimate relationships; mental health; older couples; well-being.

**Disclosure of Interest:**

None Declared

